# T cells from patients with *Candida* sepsis display a suppressive immunophenotype

**DOI:** 10.1186/s13054-016-1182-z

**Published:** 2016-01-20

**Authors:** Andrej Spec, Yuichiro Shindo, Carey-Ann D. Burnham, Strother Wilson, Enyo A. Ablordeppey, Evan R. Beiter, Katherine Chang, Anne M. Drewry, Richard S. Hotchkiss

**Affiliations:** 1 Department of Medicine, Division of Infectious Diseases, Washington University School of Medicine, 660 S. Euclid Avenue, St. Louis, MO 63110 USA; 2 Department of Anesthesiology, Washington University School of Medicine, 660 S. Euclid Avenue, St. Louis, MO 63110 USA; 3 Department of Pathology and Immunology, Division of Laboratory and Genomic Medicine, Washington University School of Medicine, 660 S. Euclid Avenue, St. Louis, MO 63110 USA; 4 Department of Surgery, Washington University School of Medicine, 660 S. Euclid Avenue, St. Louis, MO 63110 USA

**Keywords:** Bloodstream infection, *Candida*, Immunology, Sepsis, Shock, Fungal infection

## Abstract

**Background:**

Despite appropriate therapy, *Candida* bloodstream infections are associated with a mortality rate of approximately 40 %. In animal models, impaired immunity due to T cell exhaustion has been implicated in fungal sepsis mortality. The purpose of this study was to determine potential mechanisms of fungal-induced immunosuppression via immunophenotyping of circulating T lymphocytes from patients with microbiologically documented *Candida* bloodstream infections.

**Methods:**

Patients with blood cultures positive for any *Candida* species were studied. Non-septic critically ill patients with no evidence of bacterial or fungal infection were controls. T cells were analyzed via flow cytometry for cellular activation and for expression of positive and negative co-stimulatory molecules. Both the percentages of cells expressing particular immunophenotypic markers as well as the geometric mean fluorescence intensity (GMFI), a measure of expression of the number of receptors or ligands per cell, were quantitated.

**Results:**

Twenty-seven patients with *Candida* bloodstream infections and 16 control patients were studied. Compared to control patients, CD8 T cells from patients with *Candidemia* had evidence of cellular activation as indicated by increased CD69 expression while CD4 T cells had decreased expression of the major positive co-stimulatory molecule CD28. CD4 and CD8 T cells from patients with *Candidemia* expressed markers typical of T cell exhaustion as indicated by either increased percentages of or increased MFI for programmed cell death 1 (PD-1) or its ligand (PD-L1).

**Conclusions:**

Circulating immune effector cells from patients with *Candidemia* display an immunophenotype consistent with immunosuppression as evidenced by T cell exhaustion and concomitant downregulation of positive co-stimulatory molecules. These findings may help explain why patients with fungal sepsis have a high mortality despite appropriate antifungal therapy. Development of immunoadjuvants that reverse T cell exhaustion and boost host immunity may offer one way to improve outcome in this highly lethal disorder.

**Electronic supplementary material:**

The online version of this article (doi:10.1186/s13054-016-1182-z) contains supplementary material, which is available to authorized users.

## Background

Many *Candida spp.* are saprophytic fungi that occupy ecologic niches on human skin and gastrointestinal tract. In an immunocompromised host, this can lead to an opportunistic invasive infection of the skin and mucosa, or life-threatening infections of the bloodstream [1–3]. *Candida* ranges from the most common to third most common genus causing nosocomial bloodstream infections in the United States [4, 5]. Despite highly active antifungal medications, mortality remains high [6]. The mortality in *Candida* bloodstream infections approaches 40 %, higher than mortality occurring in sepsis due to most bacterial pathogens [4]. Estimates suggest that there are between 7,000 and 28,000 nosocomial *Candida* bloodstream infections per year, leading to 2,800 and 11,200 deaths per year in the United States [4, 6, 7]. A large-scale prophylaxis trial was not effective at decreasing the incidence of *Candida* bloodstream infections or improving outcome [8]. The fact that mortality from invasive fungal infections remains elevated despite the use of antimicrobial agents that are highly active against fungal pathogens, implies that defective host immunity may contribute to the persistent high mortality. Therefore, measures that augment host immunity may be fundamental to improving survival. This theory is supported by recent animal studies and a small clinical trial of patients with fungal sepsis, which demonstrated that therapies that enhance host immunity can restore immune function and, in the case of the animal studies, improve outcome [9–11].

T cell activation is carefully regulated by expression of positive and negative co-stimulatory molecules that prevent unbridled T cell function. CD28 is the classic positive co-stimulatory receptor that, acting in conjunction with the T cell receptor (TCR), induces T cells to undergo proliferation and to produce cytokines such as interferon gamma (IFN-γ) and interleukin-2 (IL-2) that are critical in controlling infection [12]. To prevent excessive T cell activation, lymphocytes also express negative co-stimulatory molecules that suppress and downregulate their function [13–16]. Programmed cell death 1 (PD-1) is a member of the B7-CD28 superfamily that functions in an inhibitory role [14–16]. During T cell activation, PD-1 is rapidly induced and expressed on the surface of CD4 and CD8 T cells where it interacts with its ligands PD-L1 and PD-L2 [13–17]. PD-L1 is expressed on both hematopoietic and non-hematopoietic cells and its expression is highly upregulated during inflammatory states [16, 18]. Activation of PD-1 by its ligands causes inhibition of many T cell functions including cytokine production and cytotoxic activity. The critical role of PD-1 in immune regulation is demonstrated by studies which showed that PD-1-null mice develop autoimmune diseases including cardiomyopathy and a lupus-like syndrome [14–16].

Increased T cell PD-1 expression occurs under conditions of chronic antigenic stimulation, such as persistent viral infections, and leads to T cell exhaustion [14–16]. These exhausted T cells are poorly functional, likely to undergo apoptosis, and ineffective thereby contributing to the chronic viral infections [14–16]. Antibodies that block PD-1 restore T cell function, increase antiviral T cell responses, and decrease viral load in certain viral infections [15, 16]. Blockade of the PD-1 pathway has also improved survival in bacterial infections. Three independent investigative teams have demonstrated that blockade of the PD-1 pathway improves survival in clinically relevant animal models of bacterial sepsis [18–20]. The potential clinical relevance of these animal studies is highlighted by recent studies showing that PD-1 overexpression on circulating T cells from patients with sepsis correlated with decreased T cell proliferative capacity, increased secondary nosocomial infections, and mortality [21, 22].

Although several studies have shown that PD-1 and PD-L1 expression is increased on T cells and antigen-presenting cells from patients with bacterial sepsis, there are no studies to date that have examined expression of PD-1 or PD-L1 on immune cells from patients with *Candida* bloodstream infections. Thus, the objective of the present study was to assess this expression. In addition to PD1 and PD-L1, we also examined immune cell expression of other important receptor/ligands that have been implicated in regulating T cell function during infection. These molecules included CD69, an early activation marker, CD28, a key positive co-stimulatory molecule, IL-7 receptor (IL-7R), which is decreased in exhausted T cells, the negative co-stimulatory molecules T cell immunoglobulin domain and mucin domain 3 (TIM-3) and B and T lymphocyte attenuator (BTLA), and CD57 which has been reported to identify poorly functional “immunosenescent” lymphocytes [23–29].

## Materials and methods

### Study design

This was a prospective observational study conducted between 2013 and 2015 and approved by the Washington University Human Research Protection Office.

### Inclusion criteria

Patients were included if they were 18 years old or older and had a blood culture positive for any *Candida* species. Potential study participants were identified by the microbiology laboratory, which notified investigators of blood cultures positive for a *Candida spp*. The control group of patients was composed of critically ill, non-septic patients (controls) who were cared for in the intensive care unit (ICU) following trauma or major surgery, but did not have fungal or bacterial sepsis.

### Exclusion criteria

The exclusion criteria were identical for the *Candida* bloodstream infections and control patients. In order to eliminate potential confounding effects of immunosuppressive medications or underlying disease on the immunophenotype of patient lymphocytes, patients with the following criteria were excluded from study: patients with human immunodeficiency virus (HIV), patients who had undergone organ or bone marrow transplantation, patients on high-dose corticosteroids (≥300 mgs/day of hydrocortisone equivalent) or other immunosuppressive medications, patients with viral hepatitis and autoimmune diseases.

### Data and sample collection

Analyses were performed on residual blood remaining after clinical hematologic testing. Because the tests were conducted on residual blood remaining in the laboratory samples (no patients had venous or arterial blood puncture) the study was granted a waiver of informed consent and approved by the Washington University Human Research Protection Office. In general, most blood samples for patients with *Candida* bloodstream infections were obtained within 24–48 hours of cultures returning positive for *Candida*. Clinical and demographic data was collected at enrollment. Patient survival versus mortality was followed for 90 days after entry into the study or until hospital discharge.

### Flow cytometry

Flow cytometric data included both the percentage of cells positive for a particular immunophenotypic marker as well as the geometric mean fluorescence intensity (GMFI), a quantitative measure of the expression of receptors or ligands expressed on a per cell basis. Antibodies for flow cytometric determinations were purchased from Biolegend (San Diego, CA, USA), BD Biosciences (San Diego, CA, USA), or eBiosciences (San Diego, CA, USA). Studies were performed on cells remaining after red blood cell lysis of diluted whole blood that had undergone antibody immunostaining as previously described [3]. Lymphocytes were identified by forward scatter (FSC) and side scatter (SSC) properties. T lymphocyte subsets were further identified by CD3+, CD4+, or CD8+ immunostaining. Additional immunostaining was performed on CD4 and CD8 T cells to identify the following:

- CD69, a marker of cell activation [9]
- CD28, a key positive co-stimulatory molecule [12],
- programmed cell death- 1 (PD-1, CD279), a negative co-stimulatory molecule that is considered a marker of cell exhaustion [10]
- programmed cell death ligand 1 (PD-L1, CD274), the ligand for PD-1 that is expressed on T cells and monocyte/macrophages, dendritic cells [10]
- BTLA, a negative co-stimulatory molecule [11]
- TIM-3, a negative co-stimulatory molecule [17]
- interleukin-7 receptor (IL-7R), signaling through IL-7 is essential for lymphocyte survival and proliferation. Decreased IL-7R expression is used to identify “exhausted” T cells [13, 14].
- CD57, a marker used to identify poorly functional “senescent” T cells [15]
- 2B4, a multifunctional receptor that may have either inhibitory or stimulatory effects on T cells [16].

### Statistical analysis

The data were analyzed using SPSS version 23 (IBM Corp., Armonk, NY, USA). Scatter plots were made using Prism (GraphPad Software Inc., San Diego, CA, USA). All continuous data were analyzed for normality using a Kolmogorov–Smirnov test with an alpha cutoff of 0.05. Normally distributed data were reported with means and standard deviations and analyzed using independent samples *t* tests. Non-normally distributed data were reported with medians and interquartile ranges and were analyzed with a Mann–Whitney *U* test. Categorical clinical data were compared using chi-square tests, or Fisher’s exact test, as appropriate. *p* ≤0.05 was considered statistically significant.

## Results

### Clinical and laboratory characteristics

During the study, 27 (62.8 %) eligible patients with *Candida* bloodstream infections that met inclusion and exclusion criteria were included. Sixteen (37.2 %) critically ill non-septic patients were recruited as controls. Baseline demographic data were similar between the two groups (Tables S1A and S1B in Additional file 1). No statistically significant differences were found between the mean age, gender, race, acute physiology and chronic health evaluation II (APACHE II) and sequential organ failure assessment (SOFA) scores, white blood cell counts, respiratory rate, heart rate, and baseline creatinine. Both of the groups had a 90-day mortality of approximately 20 %. The absolute neutrophil count was similar between patient groups, 7.23 × 10^3^ versus 6.43 × 10^3^ cells/mm^3^, respectively. The mean absolute lymphocyte count was significantly higher in patients with *Candidemia* versus control patients, i.e., 1.13 × 10^3^ cells/mm^3^ versus 0.61 × 10^3^ cells/mm^3^, respectively, ( *p* = 0.02). Both the mean absolute lymphocyte count for patients with *Candidemia* and for control, critically ill patients were below the lower limit of normal for Barnes Jewish Hospital, i.e., 1.20 × 10^3^ cells/mm^3^ (Table 1).

**Table 1:** Baseline characteristics of patients with *Candida* bloodstream infections and critically ill control patients

	CBSI N = 27 (%)		Critically ill, non-septic patients N = 16 (%)		*p* value
Mean age (±SD), years	56.9 (23.4)		58.9 (18.1)		0.19
Male gender (%)	14 (51.9)		10 (62.5)		0.46
APACHE II score (±SD)^*^	11.61 (6.7)		8.69 (4.0)		0.11
SOFA score (±SD)^*^	4.7 (4.2)		2.6 (1.8)		0.06
White blood cell count (±SD), thousand/mm^3^	11.7 (5.8)		9.9 (3.5)		0.25
Absolute lymphocyte count (±SD), thousand/mm^3^	1.13 (0.99)		0.61 (0.49)		0.02
Absolute neutrophil count (±SD), thousand/mm^3^	7.23 (5.76)		6.43 (5.32)		0.77
Heart rate (±SD), beats/min	102.2 (19.9)		109.5 (17.0)		0.23
Respiratory rate (±SD), breaths/min	23.19 (5.6)		24.4 (4.1)		0.46
Baseline creatinine (±SD), mg/dl	1.46 (1.62)		1.1 (0.85)		0.4
90-day mortality (%)	6 (22.2)		3 (18.8)		0.79
Primary diagnosis, n (%)	13 (48)	*Candida albicans*	6 (37)	Motor vehicle accident	n/a
	7 (26)	*Candida glabrata*	3 (18)	Fall	
	3 (11)	*Candida tropicalis*	2 (13)	Spinal fusion	
	3 (11)	*Candida parapsilosis*	2 (13)	Coronary artery bypass graft	
	1 (4)	*Candida dubliniensis*	2 (13)	Hemorrhage	
			1 (6)	Spinal cord injury	

^*^SOFA and APACHE II scores were calculated excluding the Glasgow Coma Score

*CBSI Candida* bloodstream infection, *SD* standard deviation, *APACHE II* acute physiology and chronic health evaluation II, *SOFA* sequential organ failure assessment

### Flow cytometry findings

**Fig. 1:**
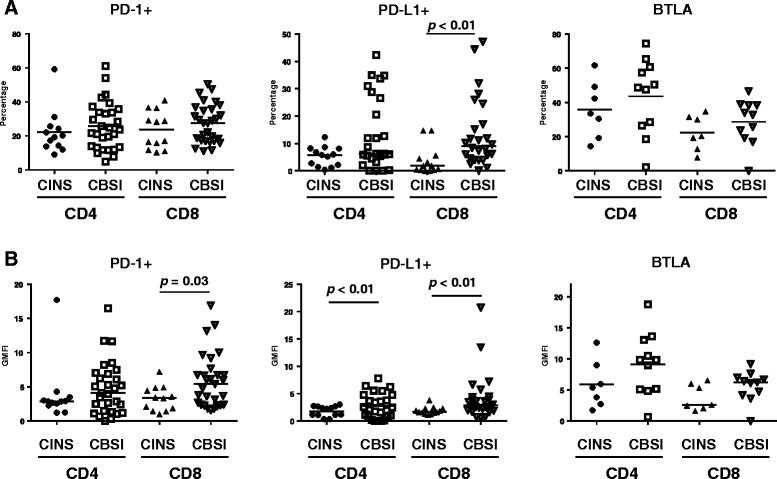
Co-inhibitory molecule expression on CD4 and CD8 T cells. PD-1, PD-L1, and BTLA are co-inhibitory molecules. The percentage of PD-L1 expression on CD8 T cells and the GMFIs of CD4 and CD8 T cells were significantly increased in patients with CBSI compared to CINS (*p* ≤0.01). Several patients with CBSI had higher percentage of PD-L1 expression on CD4 T cells than those with CINS, although there was no statistical significance between the groups (*p* = 0.161). *Horizontal lines* indicate mean or median values. *Asterisks* indicate significant differences between CINS and CBSI (^*^ *p* ≤0.05 and ^**^ *p* ≤0.01). *BTLA* B and T lymphocyte attenuator, *CBSI Candida* bloodstream infection, *CINS* critically ill non-sepsis, *GMFI* geometric mean fluorescence intensity, *PD-1* programmed cell death 1, *PD-L1* programmed cell death ligand 1

**Fig. 2:**
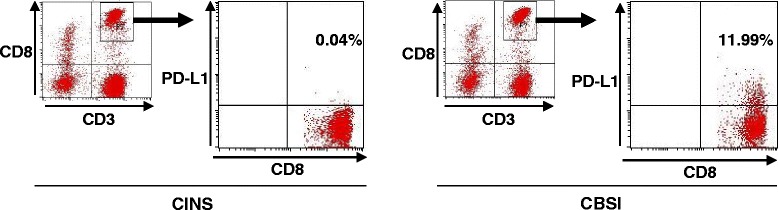
PD-L1 expression on CD8 T cells in CINS and CBSI. Representative flow cytometric findings in patients with CINS and CBSI are shown. The percentage of PD-L1-positive CD8 T cells was higher in CBSI than in CINS. *CBSI Candida* bloodstream infection, *CINS* critically ill non-sepsis, *PD-1* programmed cell death 1, *PD-L1* programmed cell death ligand 1

Although there was no difference in the percentage of CD8 T cells that were positive for PD-1 in patients with *Candidemia* versus control patients, patients with *Candidemia* did have an increase in the geometric mean fluorescence intensity (GMFI), indicating that the cells which were positive for PD-1 had an increase in the number of PD-1 molecules on a per cell basis; (*p* <0.05), (Fig. 1). For some signaling molecules or hormones, the number of cell receptors (indicated by the MFI) is an important determinant of the cell response. There were no differences in either the percentage of cells positive for PD-1 or the MFI for PD-1 from CD4 T cells of patients with *Candidemia* versus control patients. For PD-L1, both the percentage of CD8 T cells positive for PD-L1 as well as the MFI of the cells that were positive for PD-L1 was increased in patients with *Candidemia* versus control patients; *p* <0.01, (Figs. 1 and 2). Compared to control patients, the MFI for PD-L1 was increased in CD4 T cells from patients with *Candidemia*; *p* <0.05. There was no difference in the percentage of CD4 T cells that were positive for PD-L1 in patients with *Candidemia* versus control patients, (Fig. 1). These results, showing a more prominent effect of fungal sepsis on expression of PD-1 and PD-L1 on CD8 T cells compared to CD4 T cells, have been observed in previous studies of patients with bacterial sepsis [30].

**Fig. 3:**
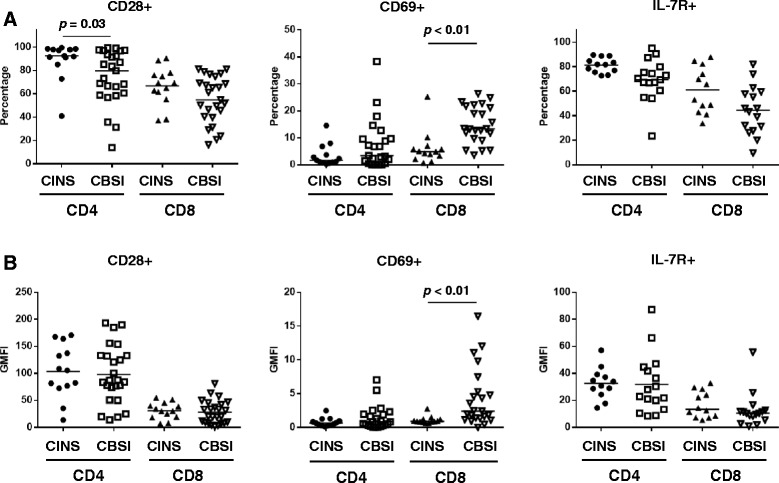
Co-stimulatory molecule expression and markers related to cell activation on CD4 and CD8 T cells. CD28 is a co-stimulatory molecule that is necessary for optimal T cell activation. CD69 is marker of cellular activation. IL-7R activation leads to T cell activation and proliferation. CD28 expression on CD4 T cells was significantly decreased in patients with CBSI, compared to those with CINS. In CD8 T cells, both the percentage and GMFI for CD69 were increased in CBSI compared to CINS. On the other hand, the percentage and GMFI of the IL-7R tended to be lower in CD4 T cells from CBSI versus CINS patients. However, this difference did not reach statistical significance, *p* = 0.07 and *p* = 0.08 respectively. *Horizontal lines* indicate mean or median values. *Asterisks* indicate significant differences between CINS and CBSI (^*^ *p* ≤0.05 and ^**^ *p* ≤0.01). *CBSI Candida* bloodstream infection, *CINS* critically ill non-sepsis, *GMFI* geometric mean fluorescence intensity, *IL-7R* interleukin-7 receptor

The percentage of CD8 T cells positive for the early activation marker CD69 was increased in patients with *Candidemia* versus control patients, 13.36 % versus 4.85 %, respectively, (Fig. 3), (*p* <0.01). Similarly, the MFI of CD69 in CD8 T cells from patients with *Candidemia* was also increased compared to control patients; *p* <0.01. No differences in CD69 expression were observed in patients with *Candidemia* versus control patients for CD4 T cells.

In contrast to the increase in *inhibitory* receptor/ligands noted in CD8 T cells, there was a significant decrease in the percentage of CD4 T cells expressing CD28, a major positive *co-stimulatory* molecule, in patients with *Candidemia* compared to controls, 79.8 % versus 92.6 %, respectively; *p* <0.05, (Fig. 3). There were no statistical differences for either the percentage of cells positive for CD28 or the MFI for CD28 in CD8 T cells from the two groups of patients.

**Fig. 4:**
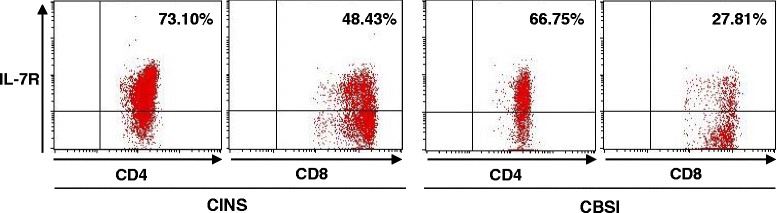
Interleukin 7 receptor expression on CD4 and CD8 T cells - differences between CINS and CBSI. Representative flow cytometric findings in patients with CINS and CBSI are shown. The percentages of IL-7R-positive CD4 and CD8 T cells were lower in CBSI than in CINS. *CBSI Candida* bloodstream infection, *CINS* critically ill non-sepsis, *IL-7R* interleukin- 7 receptor

**Fig. 5:**
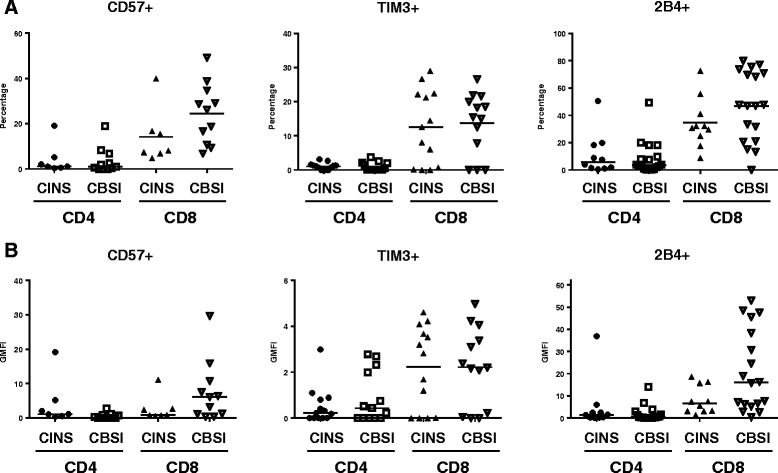
CD marker expression on CD4 and CD8 T cells related to senescence and inhibitory or stimulatory effects. CD57 is a marker that is used to identify senescent, poorly functional T cells. TIM-3 is a co-inhibitory molecule that impairs T cell function. 2B4 (CD244) is a dual-functional receptor that may have either inhibitory or stimulatory effects on T cells depending upon other stimuli and environmental settings. *Horizontal lines* indicate mean or median values. *Asterisks* indicate significant differences between CINS and CBSI (^*^ *p* ≤0.05 and ^**^ *p* ≤0.01). *CBSI Candida* bloodstream infection, *CINS* critically ill non-sepsis, *TIM-3*T cell immunoglobulin domain and mucin domain 3

Decreased expression of IL-7 receptor (IL-7R) is characteristic of poorly functional CD4 and CD8 T cells that result from chronic antigenic stimulation [18, 19]. Although the difference did not quite reach statistical significance, there was a trend toward a decreased percentage of CD4 T cells positive for the IL-7R and decreased MFI in patients with *Candidemia* compared to controls, *p* = 0.08 and *p* = 0.07 respectively (Figs. 3 and 4). There were no statistical differences in either the percent positivity or the MFI for other markers of T cell exhaustion, i.e., BTLA, CD57, TIM-3, or 2B4 for either CD4 or CD8 T cells in the two groups of patients, (Figs. 1 and 5).

## Conclusions

The incidence of *Candida* bloodstream infection is increasing and *Candida* is currently one of the most common nosocomial bloodstream infections in many intensive care units (ICUs) [1, 2, 4, 5, 20, 21]. The ability of the host to survive a *Candida* bloodstream infection requires a well-coordinated response by the innate and adaptive immune system, both of which are frequently impaired in patients with fungal sepsis [22–24]. Many of the patients acquiring fungal sepsis are immunosuppressed due to underlying malignancies and treatment with chemo or radiation therapy. Patients undergoing bone marrow or solid organ transplantation are also immunosuppressed and have a high incidence of fungal infection. There is increasing recognition that many ICU patients who are presumed to be immunocompetent also acquire invasive fungal infections. Many of these patients are elderly individuals whose immune system is impaired due to “immunosenescence” [31]. Additionally, ICU patients with bacterial sepsis have impaired immunity due to a variety of factors including: loss of immune effector cells, increased immunosuppressive cells including T regulatory cells and myeloid-derived suppressor cells, and T cell exhaustion [22]. Patients in the ICU also may require central venous lines, arterial lines, and Foley catheters, which compromise the body’s protective barrier. In this regard, the majority of the fungal infections in the present study were felt to be related to invasive lines (Additional file 1: Table S1A) rather than to parenchymal infections. Also, the present study excluded patients who had undergone bone marrow or stem cell transplantation, HIV patients, and patients on high-dose immunosuppressive medication. These factors may explain the relatively low mortality in the present study (22 %) compared to higher mortality figures reported in other studies [4].

The fact that many patients with fungal sepsis die despite being treated with drugs to which the fungal pathogens are sensitive suggests that impaired host immunity is a factor in the poor survival seen in fungal sepsis. Thus, enhancing host immunity may improve outcome. This hypothesis is supported by studies of interleukin-7 (IL-7), a pleiotropic cytokine that enhances T cell function [10, 32–35]. Using a mouse model of fungal sepsis, our group demonstrated that IL-7 improved IFN-γ production, decreased apoptosis, and increased survival in mice with *Candida* sepsis [14]. Furthermore, a report in which investigators treated patients with invasive fungal infections and decreased HLA-DR expression, a measure of impaired immunity, with IFN-γ immunotherapy demonstrated an improvement in patient immune function [9]. Thus, immunoadjuvant therapy is moving forward in fungal sepsis and the ability to identify those patients who are good candidates for immunotherapy is needed.

To our knowledge, this is the first study to investigate the immunophenotype of lymphocytes of patients with *Candida* bloodstream infection. The present results are consistent with T cell exhaustion, a condition that occurs following chronic antigenic stimulation in which T cells become poorly functional with reduced cytokine production, decreased proliferative capacity, and are prone to undergo apoptotic cell death. An increase in T cell expression of the inhibitor receptor PD-1 and its ligand, PD-L1 are essential for mediating T cell exhaustion [16, 17, 19, 25, 26]. In this study, CD8 T cells from patients with *Candida* bloodstream infection had an increase in both the percentage of cells positive for PD-L1 and in the MFI (a measure of the number of receptors or ligands on a per cell basis) for PD-L1 compared to controls (Figs. 1 and 2). Similarly, CD4 T cells from patients with *Candida* bloodstream infection had an increase in the MFI for PD-1 expression compared to controls.

Another cell phenotypic marker that is consistent with exhausted T cells is decreased T cell IL-7R expression [24]. Decreased IL-7R results in decreased concentrations of Bcl-2, an anti-apoptotic protein that is essential for T cell survival. The present results showed a non-statistically significant trend toward a decrease in the percentage of CD4 T cells positive for IL-7R (*p* = 0.08) and in the MFI (*p* = 0.07) in patients with *Candida* bloodstream infection compared to controls. Although there were no differences in BTLA, TIM-3, or 2B4, other markers of T cell exhaustion, in patients with *Candida* bloodstream infection versus controls, cellular expression of these markers is not as frequently abnormal as expression of PD-1 or PD-L1 in T cell exhaustion [14].

In addition to markers of T cell exhaustion, patients with *Candida* bloodstream infection also had another potentially important finding consistent with an impaired T cell response to pathogens, i.e., a decrease in the percentage of CD4 T cells expressing CD28. CD28 is a key co-stimulatory molecule that is essential for T cell activation and survival [17]. Stimulation through CD28 activates signaling pathways that result in production of various cytokines that help to combat infectious pathogens. Previous work from our group has shown that expression of CD28 is decreased in both animals and patients with sepsis [10, 22]. In this regard, CD4 T cells from animals with sepsis that were treated with IL-7 had an increase in CD28 expression and improvement in T cell function.

In contrast to the high mortality, often approaching 40 %, which typically is associated with invasive fungal infections, the mortality in the present study was only 22 %. There are several reasons for the relatively low mortality in the present study. First, the present study excluded patients who underwent organ or bone marrow transplantation. These patients have a high incidence of fatal fungal infections because of their severely impaired immune systems. Second, we also excluded acquired immunosuppressive deficiency syndrome (AIDS) patients and patients who were taking high-dose corticosteroids ((≥300 mgs/day of hydrocortisone equivalent) or other immunosuppressive medications. These patients are also more likely to succumb to fungal infections because of their inability to mount an effective immune response.

This study has a number of limitations. First, although this study showed that lymphocytes from patients with fungal bloodstream infections have phenotypic markers consistent with immunosuppression and T cell exhaustion, no cell functional studies were performed. Thus, we do not know the degree or intensity of T cell impairment. Second, these findings do not establish a causal link between markers of T cell exhaustion and increased morbidity or mortality in patients with fungal sepsis. Although studies do demonstrate a correlation of increased T cell exhaustion and decreased outcomes in sepsis, no such studies exist for patients with fungal sepsis. It is important to note, however, that previous studies from our group showed that therapy with anti-PD-1 and anti-PD-L1 antibodies reversed T cell dysfunction and improved survival in both primary candidiasis and in a two-hit model of fungal infection [11]. These studies do provide support for the hypothesis that dysfunctional T cells are a key pathologic factor in lethal fungal infections.

A second limitation of the study is that we are not able to determine a reason for the difference in the phenotypic response of CD4 and CD8 T cells during fungal infection. The present findings show a greater effect of fungal sepsis to increase PD-1 and PD-L1 expression (either on the percentage positive and/or MFI for PD-1 or PD-L1) on CD8 T versus CD4 T cells. We have previously reported differential effects of bacterial sepsis on PD-1 and PD-L1 in CD8 versus CD4 T cells [11, 22, 30]. Although there is no clear explanation for this different cellular effect, it may be related to the unique functions of CD4 versus CD8 T cells, i.e., CD4 T cells having a “helper” function to activate other immune cells while CD8 T cells act to eliminate pathogens by cell killing.

In conclusion, the present results demonstrate that T cells from patients with *Candida* bloodstream infection have a phenotype consistent with an immunosuppressive “exhaustive” state. In the future, examination of these flow cytometric markers may be useful in identifying patients with fungal sepsis who have impaired immunity and thus are candidates for trials of agents which boost host immunity.

## Key messages

- Patients with *Candida* bloodstream infections exhibit an immunosuppressed phenotype.
- This phenotype appears to be mediated through the PD-1 axis.

## Additional file

Additional file 1: Table S1A. Characteristics of patients with *Candida* infection (n = 27). Table S1B. Characteristics of critically ill control patients (n = 16). (DOC 41 kb)

## Abbreviations

BTLA: B and T lymphocyte attenuator
(G)MFI: (Geometric) mean fluorescence intensity
ICU: Intensive care unit
IFN-γ: Interferon gamma
IL-2: Interleuken-2
IL-7: Interleuken-7
IL-7R: IL-7 receptor
PD-1: Programmed cell death 1
PD-L1: Programmed cell death ligand 1
PD-L2: Programmed cell death ligand 2
TCR: T cell receptor
TIM-3: T cell immunoglobulin domain and mucin domain 3
