# Supplementary material for: T cells from patients with Candida sepsis display a suppressive immunophenotype
Source: Crit Care. 2016 Jan 20;20:15. doi: 10.1186/s13054-016-1182-z (PMC4719210; doi:10.1186/s13054-016-1182-z)
Supplement: Additional file 1: Table S1A. — Characteristics of patients with Candida infection (n = 27). Table S1B. Characteristics of critically ill control patients (n = 16). (DOC 41 kb) [file 13054_2016_1182_MOESM1_ESM.doc]

Table 1A. Characteristics of Patients with Candida Infection (n=27)

| **Mean Age (±SD), years** | **56.9 (23.4)** |
| --- | --- |
| **Male Gender (%)** | 14 (51.9) |
| **APACHE II Score (±SD)*** | 11.61 (6.7) |
| **SOFA Score (±SD)*** | 4.7 (4.2) |
| **White Blood Cell Count (±SD), Thousand/mm^3^** | 11.7 (5.8) |
| **Absolute Lymphocyte Count (±SD), Thousand/mm^3^** | 1.13 (0.99) ** |
| **Absolute Neutrophil Count (±SD), Thousand/mm^3^** | 7.23 (5.76) |
| **Heart Rate(±SD), beats/min** | 102.2 (19.9) |
| **Respiratory Rate (±SD), breaths/min** | 23.19 (5.6) |
| **Baseline Creatinine (±SD), mg/dl** | 1.46 (1.62) |
| **90 Day Mortality (%)** | 6 (22.2) |
| **Site of Infection, n (%)** | Fungal line infection, 20 (74)  Fungal peritonitis, 5 (18)  Fungal pyelorephritis, 1 (4)  Fungal wound infection, 1 (4) |
| **Microbiology, n (%)** | *Candida albicans,* 13 (48)  *Candida glabrata*, 7 (26)  *Candida tropicalis*, 3 (11)  *Candida parapsilosis*, 3 (11)  *Candida dubliniensis*, 1 (4) |
| **Primary Diagnosis, n** | Endocarditis, 1  Osteomyelitis, 1  Pyelonephritis, 2  Peritonitis, 6  Pneumonia/respiratory failure, 3  Line infection, 7  Encephalitis/coma, 2  Congestive heart failure, 3  Colitis/diarrhea, 2  Sickle cell crisis, 1 |
| **Co-morbidities** | Diabetes, 10  Heart disease, 5  Morbid obesity, 3  Neurologic, 2  Renal, 5  COPD, 6  GI - enterocutaneous fistula, 2  GI – pancreatitis, 1 |

*SOFA and APACHE II scores were calculated excluding the Glasgow Coma Score
SD, Standard Deviation

** significantly different from critically-ill control patients, p=0.02

COPD, chronic obstructive pulmonary disease

GI, gastrointestinal

Table 1B. Characteristics of Critically-Ill Control Patients (n=16)

|  |  | **p value** |
| --- | --- | --- |
| **Mean Age (±SD), years** | 58.9 (18.1) | 0.19 |
| **Male Gender (%)** | 10 (62.5) | 0.46 |
| **APACHE II Score (±SD)*** | 8.69 (4.0) | 0.11 |
| **SOFA Score (±SD)*** | 2.6 (1.8) | 0.06 |
| **White Blood Cell Count (±SD), Thousand/mm^3^** | 9.9 (3.5) | 0.25 |
| **Absolute Lymphocyte Count (±SD), Thousand/mm^3^** | 0.61 (0.49) | 0.02 |
| **Absolute Neutrophil Count (±SD), Thousand/mm^3^** | 6.43 (5.32) | 0.77 |
| **Heart Rate(±SD), beats/min** | 109.5 (17.0) | 0.23 |
| **Respiratory Rate (±SD), breaths/min** | 24.4 (4.1) | 0.46 |
| **Baseline Creatinine (±SD), mg/dl** | 1.1 (0.85) | 0.4 |
| **90 Day Mortality (%)** | 3 (18.8) | 0.79 |
| **Primary Diagnosis, n (%)** | Motor Vehicle Accident, 6 (37)  Fall, 3 (18)  Spinal fusion, 2 (13)  Coronary artery bypass graft, 2 (13)  Hemorrhage,2 (13)  Spinal cord injury,1 (6) | n/a |

*SOFA and APACHE II scores were calculated excluding the Glasgow Coma Score
SD, Standard Deviation
